# Degradable Gel for Temporary Plugging in High Temperature Reservoir and Its Properties

**DOI:** 10.3390/gels10070445

**Published:** 2024-07-05

**Authors:** Fan Yang, Jinhua Liu, Renjing Ji, Xiaorong Yu, Huan Yang, Gaoshen Su

**Affiliations:** 1 State Key Laboratory of Shale Oil and Gas Enrichment Mechanisms and Effective Development, Beijing 102206, China; yangfan.sripe@sinopec.com (F.Y.); liujinhua.sripe@sinopec.com (J.L.); 2 Sinopec Research Institute of Petroleum Engineering Co., Ltd., Beijing 102206, China; 3 College of Chemistry and Environmental Engineering, Yangtze University, Jingzhou 434023, China; 2023710235@yangtzeu.edu.cn (R.J.); yanghuan@yangtzeu.edu.cn (H.Y.); sugaoshen@163.com (G.S.); 4 HuBei Engineering Research Centers for Clean Production and Pollution Control of Oil and Gas Fields, Jingzhou 434023, China

**Keywords:** degradable gel, unstable crosslinker, viscoelastic properties, thermal stability, core damage

## Abstract

Although various degradable gel materials have been developed for temporary plugging in oil fields, they often degrade too quickly in high-temperature environments. To address this issue, an unstable crosslinker was synthesized to prepare a high-temperature degradable gel. This gel does not degrade excessively fast at high temperatures. Temperature and crosslinker concentration are the primary factors influencing gel degradation time, followed by monomer and initiator concentrations. Increased temperature and decreased crosslinker concentration both reduce degradation time, which can be adjusted within the range of 90–130 °C by varying the crosslinker concentration. The molecular structure and thermal stability of the degradable gel were analyzed using FTIR, ^13^C NMR, and TG. Furthermore, the viscoelastic properties, compressive performance, plugging performance, and core damage performance of the gel were evaluated. Within the test range of 0.1–1000 Pa, the storage modulus is higher than the loss modulus. The gel prepared at 130 °C exhibited a compressive stress of 0.25 MPa at 50% strain. The plugging pressure of the gel in sand-filled tubes with varying permeabilities (538.2–2794.1 mD) exceeded 15 MPa while maintaining a core damage rate below 5%. SEM analysis indicated that the degradation mechanism of the gel may involve the collapse of its three-dimensional network structure due to the hydrolysis of amide groups in the crosslinker. The viscosity of the degradation liquid was below 11 mPa·s, enabling it to be brought back to the surface with the formation fluid without the need for further breaking operations.

## 1. Introduction

Since the onset oil and gas exploitation and development, there have been countless accidents caused by well leakage, such as well wall instability, formation collapse, blowout, and a series of other problems; these problems have brought about huge economic losses for oil and gas development [1,2,3,4,5,6]. Therefore, many scholars have researched the drilling leakage mechanism and have developed a variety of plugging materials. The main types are as follows: bridging plugging materials, flexible and elastic plugging materials, expansive plugging materials, gel plugging materials, and so on [7,8,9,10,11,12,13]. The gel plugging material has been widely applied in the field and its advantages are obvious. First, the scope of application is wide: regardless of pore-type leakage or crack-type leakage, gel plugging materials can be applied [14]. Gel plugging materials also exhibit good degradation ability, allowing for unblocking through processes such as bio-degradation, thermal degradation, oxidative degradation, and other methods. This characteristic is beneficial for reservoir protection [15,16,17]. Li et al. [18] prepared a high temperature high strength anti-H_2_S gel valve; the gel valve was completely broken after adding 10% strong oxidizing gel breaker for 60 min and the residual liquid could be discharged back to the ground without affecting the subsequent normal operation. Zheng et al. [19] evaluated the chemical gel breaker for ultra-high strength gels in multistage networks, and they found that the acid could reduce the volume of the gel and decrease the strength of the gel, increase the contact area between the breaker and the gel, and significantly improve the breaking effect.

For the protection of the reservoir and for simultaneously simplifying the construction process, some scholars have prepared degradable plugging gels. For example, Wang et al. [20] prepared a novel degradable hydrogel by free radical aqueous solution polymerization, which can be used for temporary plugging in the acidic fracturing and can be degraded in acid, salt solution, and water. Ji et al. [21] prepared a self-breaking gel valve synthesized from acrylamide and degradable crosslinker for sealing off formation pressure and studied the self-breaking mechanism of the gel valve in depth. Wang et al. [22] synthesized a self-degradable gel with high adhesion by using acrylamide, acrylic acid, and an unstable crosslinker as raw materials. Due to the presentation of the unstable crosslinker, this gel does not require additional injection of chemical gel breakers at the end of the operation. The degradation time of the gel can be controlled by designing the formulation to achieve rapid degradation of the gel.

In the above studies, the gels were degraded due to the hydrolysis of the unstable ester groups. Generally, the optimal temperature for ester hydrolysis is between 50–80 °C, so the experimental temperatures in the above studies were all lower than 100 °C. However, with the deepening of drilling operations, high downhole temperatures limit the development of plugging materials, especially gel plugging materials [23,24,25,26]. The temperature resistance of gel plugging materials is poor, and at high temperatures, gels are easily thermally broken down leading to excessive degradation [27]. To solve the problem of poor temperature resistance of gels, current research has focused on enhancing the temperature resistance of gels by introducing a temperature resistant component [28,29,30]. To satisfy the controlled and regulated degradation of the gels at high temperatures, amides with lower hydrolysis reactivity than ester groups were chosen to prepare a crosslinker that could resist high temperatures but at the same time could be hydrolyzed.

In this paper, a high-temperature unstable crosslinker (N, N′-butylbis[(3-acrylamidopropyl) dimethylammonium bromide]) was synthesized, and a degradable gel was prepared using this crosslinker. The effects of various preparation conditions, including monomer concentration, crosslinker concentration, initiator concentration, and temperature, on the degradation time of the degradable gel were investigated through orthogonal experiments. A series of characterization analyses were carried out on the degradable gel. The compression properties, plugging performance, and core damage of the degradable gel were subsequently studied. Finally, the degradation properties of the degradable gel were examined. The findings enrich the selection of plugging materials suitable for high-temperature reservoirs under pressure operation.

## 2. Results and Discussion

### 2.1. Analysis of Orthogonal Experiment Results

The results of the orthogonal experiment are presented in Table 1. $k_{1}$, $k_{2}$, and $k_{3}$ represent the influence of each level on the experimental results for a specific factor. When the factor is at level i (i = 1, 2, 3), ki is calculated by dividing the sum of experimental results by the number of experiments at that level. The range of variation in the influence of a factor on the experimental results is denoted by R, calculated using Equation (1). The larger the R value, the more significant the influence of this factor on the experimental results. By comparing the factors of R value, we can identify which factors have a greater effect on the experimental results.

(1) $$R=\max\;(k_{1},k_{2},k_{3})-\min\;(k_{1},k_{2},k_{3})$$

Among the four experimental factors, the order of the R of each factor for the degradation time of the gel is: temperature > crosslinker concentration > monomer concentration > initiator concentration. Based on the results of the orthogonal experiments, it can be seen that the temperature has the greatest effect on the degradation time of the degradable gel, followed by the crosslinker concentration. The higher the temperature, the shorter the degradation time. The properties of the functional groups on the polymer chains of the degradable gel are altered by the increase in temperature, and the ends of the chains are contracted and curled. In addition, the junction between the crosslinker and the gel may break, resulting in a lower crosslink density of the degradable gel and a larger three-dimensional network framework space, which is more prone to degradation into individual macromolecules. The degradation time of gels at high temperatures is dramatically shortened, resulting in limited field applications and failure to achieve the desired sealing time.

**Figure 1:**
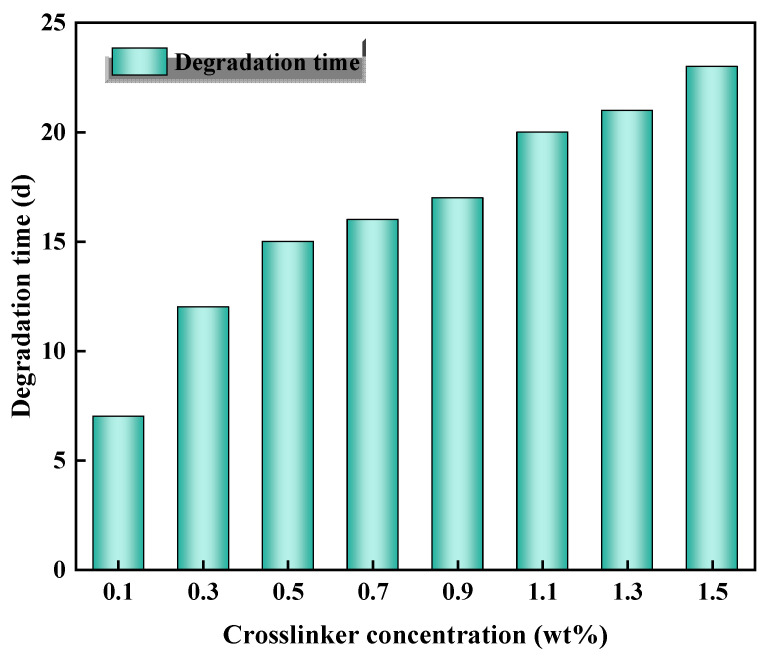
Effect of crosslinker concentration on degradation time of the gel.

Fortunately, the degradation time can be extended by other methods. The gel degradation time was determined by altering the crosslinker concentration at a monomer concentration of 10 wt% and an initiator concentration of 0.02 wt%. The results depicted in Figure 1 show an increase in degradation time from 7 days to 23 days as the crosslinker concentration rose from 0.1 wt% to 1.5 wt%. Crosslinkers also have a significant effect on the degradation time, with the larger the dosage, the longer the degradation time of the degradable gel. The crosslinker plays a crucial role in connecting polymer chains to create a network structure. As the concentration of crosslinker increases, more crosslinking points form in the system, resulting in a denser three-dimensional gel network. This denser structure compresses the space for water molecules inside the gel, ultimately leading to a reduction in water molecules. This reduction negatively impacts the hydrolysis of the amide group, thereby prolonging its degradation time. The degradation time can be adjusted at 90–130 °C (5–25 d) by changing the concentration of crosslinker dosage to meet the needs of on-site construction.

### 2.2. FTIR Analysis

**Figure 2:**
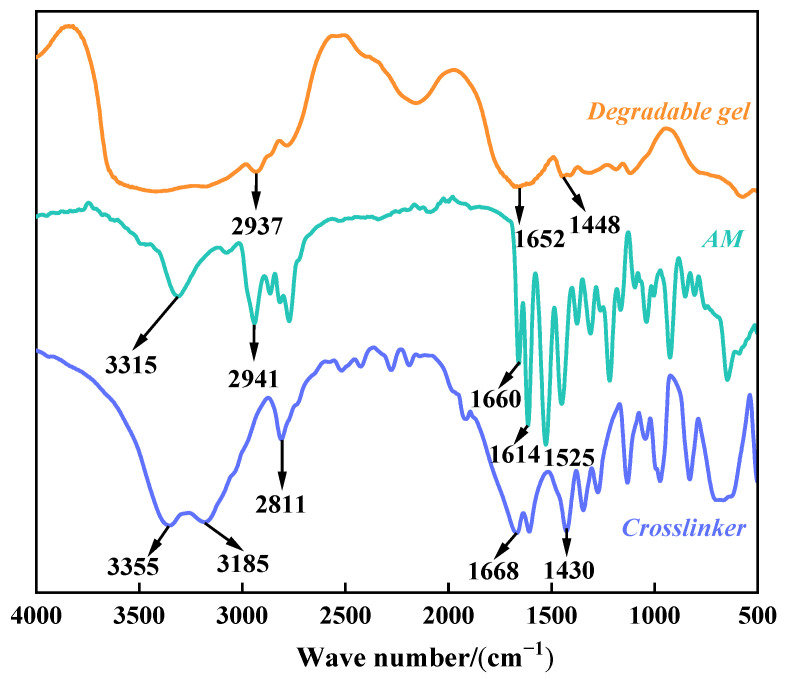
Infrared spectra of AM, crosslinker and degradable gel.

The infrared spectra of the acrylamide (AM), crosslinker, and degradable gel are shown in Figure 2. In the spectrum of AM, strong absorption peaks of C=O and C=C at 1668 cm^−1^ are observed. In the infrared spectra of crosslinker, 3315 cm^−1^ is the unsymmetrical stretching vibration absorption peak of N-H in the amide group, 2941 cm^−1^ is the -C-H stretching vibration, the stretching vibration of C=O and C=C at 1660 cm^−1^, the bending vibration of N-H at 1614 cm^−1^, and the stretching vibration absorption peak of C-N at 1525 cm^−1^. In the spectrum of the degradable gel, 3195 cm^−1^ is the unsymmetrical stretching vibration absorption peak of N-H, 2937 cm^−1^ is the -C-H stretching vibration, the C=O stretching vibration at 1652 cm^−1^, and 1448 cm^−1^ is the C-N stretching vibration absorption peak. By comparing the absorption peaks of the infrared spectra, it can be seen that the absorption peak of the gel near 1660 cm^−1^ is weakened in intensity compared with that of the monomer and the crosslinker and becomes a broad peak, indicating that the formation of the degradable gel is realized by destroying the C=C double bond to realize the crosslinking polymerization [21,22].

### 2.3. ^13^C NMR Analysis

**Figure 3:**
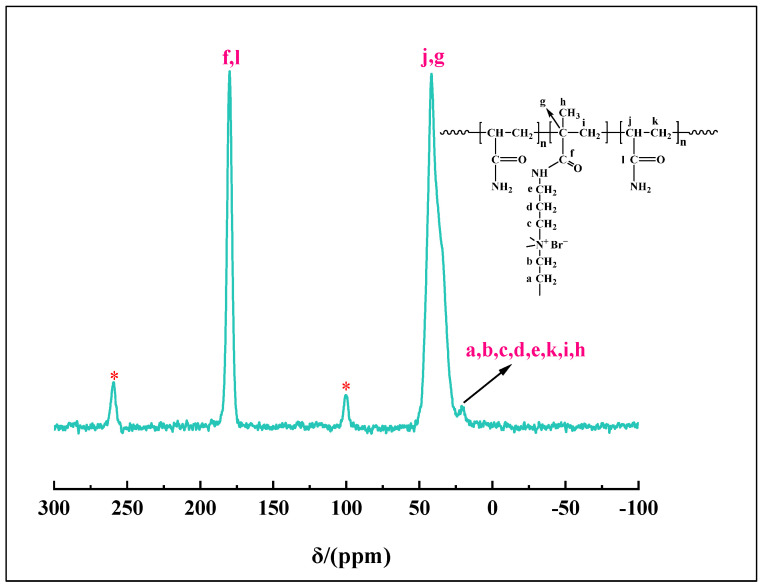
^13^C NMR spectrum of degradable gel (*: spinning sideband).

Figure 3 presents the ^13^C nuclear magnetic resonance (^13^C NMR) spectrum of the degradable gel. The letters a-k represent different positions of carbon. The chemical shift at 179.9 ppm is attributed to the amide carbonyl carbons C_f_ (C=O) and C_l_ (C=O) of the degradable gel. The chemical shift of the degradable gel at 41.7 ppm is attributed to C_j_ (CH) and C_g_ (-C-). The signal at 20.7 ppm can be assigned to C_a_ (CH_2_), C_b_ (CH_2_), C_c_ (CH_2_), C_d_ (CH_2_), C_e_ (CH_2_), C_k_ (CH_2_), C_i_ (CH_2_), C_h_ (CH_3_) [31]. In concordance with FTIR analysis, ^13^C NMR analysis likewise confirmed that the degradable gel prepared in this study was copolymerized with AM and crosslinker.

### 2.4. TG Analysis

**Figure 4:**
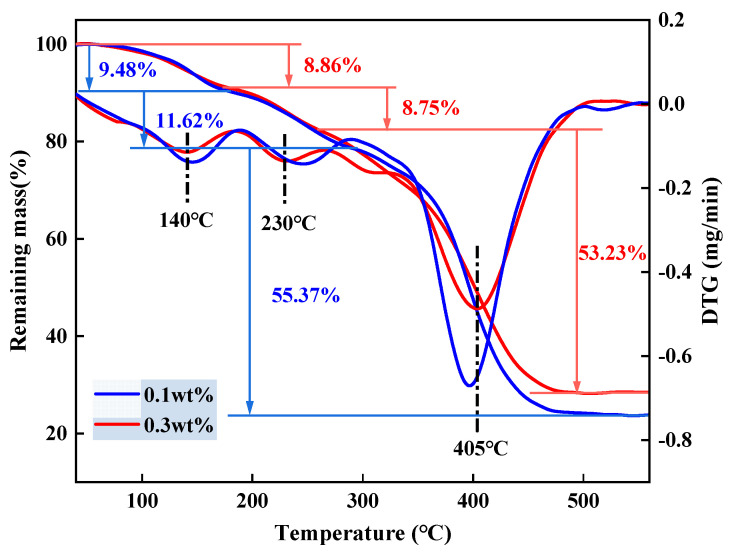
TG curves and DTG curves of the degradable gel.

It was mentioned in 2.1 that the concentration of crosslinker has a noticeable effect on the degradation properties of the gel, and in this section, the thermal stability of the gel with different crosslinker concentration was further investigated. The test samples were prepared with 10 wt% of monomer, 0.02 wt% of initiator, and a temperature of 90 °C. The concentration of crosslinker was 0.1 wt% and 0.3 wt%. The thermal analysis (TG) curves of the two degradable gels are shown in Figure 4. The degradable gels with different crosslinker concentrations have very similar shapes of TG curves, which can be divided into three segments. The first stage is the loss of water within the degradable gel, including free and weakly bound water. The loss of mass is minimized in this stage because the gel has been dried prior to testing and most of the water has been removed. The second stage is the pyrolysis of the amide groups in the degradable gel and the breaking of the branched chains. The third stage is the breakage of the polymer main chain and the crosslinked network structure, and this stage of the gel has the greatest mass loss. The final mass residue of the degradable gels with a crosslinker concentration of 0.3 wt% was 29.16%; the final mass residue of the degradable gels with a crosslinker concentration of 0.1 wt% was 23.53%. The major breakdown of the gels started at 140 °C and was completed at about 500 °C. After 400 °C, the weight loss of the degradable gel with high crosslinker concentration was significantly reduced, which was attributed to the crosslinking between the linear polymer chains and the crosslinker molecules, which resulted in a denser internal structure of the degradable gel and provided excellent thermal stability. Thermal stability analysis showed that the degradable gel has good thermal stability and can be used at temperatures lower than 140 °C. In addition, increasing the amount of crosslinker can result in more crosslinked structures, thus improving the thermal stability of the gel.

### 2.5. Viscoelastic Properties of Degradable Gel

**Figure 5:**
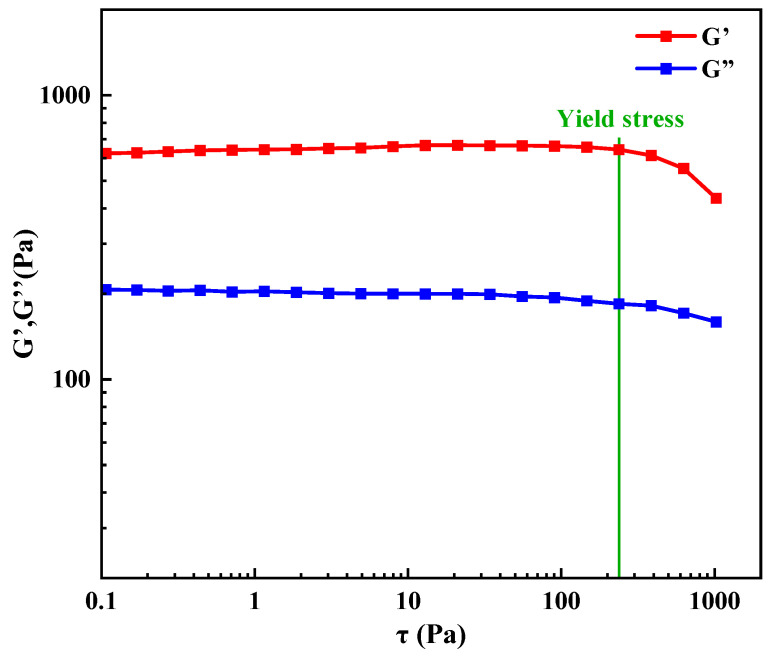
Storage modulus $G^{\prime \prime }$ and loss modulus $G^{\prime \prime }$ of degradable gel.

The storage modulus ($G^{\prime }$) and the loss modulus ($G^{\prime \prime }$) have a significant effect on the strength and the plugging effect of the degradable gel. The material is viscoelastic when $G^{\prime }$ and $G^{\prime \prime }$ are of similar magnitude [32]. The mathematical definitions of $G^{\prime }$ and $G^{\prime \prime }$ are given below Equations (2) and (3).

(2) $$G^{\prime }=\left(\frac{\sigma_{0}}{\varepsilon_{0}}\right)\times \cos\left(\delta \right)$$

(3) $$G^{\prime \prime }=\left(\frac{\sigma_{0}}{\varepsilon_{0}}\right)\times \sin\left(\delta \right)$$

where *σ*_0_ is the amplitude of the stress, *ε*_0_ is the amplitude of the strain, and *δ* is the phase angle. Viscoelasticity is a property between a solid and a liquid, i.e., it has both the elastic property of a solid and the flow property of a liquid. A larger the viscoelastic modulus of the gel indicates that its three-dimensional mesh structure is denser and more solid, with stronger anti-shear properties and better plugging effect. Therefore, it is necessary to study the viscoelastic properties of gels applied to the oilfield. The results of the viscoelastic properties of the degradable gel are shown in Figure 5. The stress scanning test also gives information about the pattern of change in the viscoelasticity of the gel, where the transition from elastic to viscous behavior is due to the deformation of the network structure within the degradable gel. There is a critical stress in the scanning test experiment, the yield stress, which is the minimum force at which the material begins to deform. When the stress on the gel is below the yield stress, $G^{\prime }$ remains relatively constant. However, once the stress surpasses the yield stress, $G^{\prime }$ starts to decrease, indicating deformation in the network structure of the gel and a transition from elastic behavior to viscous. The results depicted in Figure 5 show that the degradable gel exhibits characteristics of a semi-solid material with a yield stress of 237 Pa. Throughout the test range of 0.1–1000 Pa, $G^{\prime }$ consistently surpasses $G^{\prime \prime }$, indicating the gel’s preference towards elastic behavior. Moreover, the degradable gel demonstrates high strength in grade I post-gelation. Under compression, the gel experiences elastic deformation akin to a solid, attributed to its dense three-dimensional network structure that confers deformation resistance and pressure-bearing capabilities.

### 2.6. Compressive Performance of Degradable Gel

**Figure 6:**
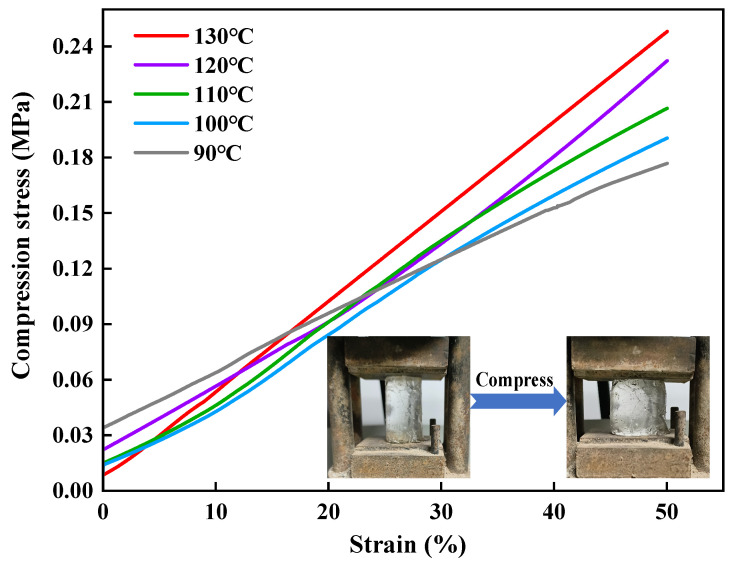
Compressive stress-strain curves of degradable gels at 90–130 °C.

The compression stress-strain test is to observe the force and deformation of degradable gel under compression so as to judge the compression performance. Figure 6 shows the compression test results of the series of degradable gels at 50% fixed strain. It can be seen that there is no obvious difference in the shape of the stress-strain curves of the degradable gels prepared at different temperatures, and the degradable gels showed excellent compression elasticity. When the degradable gel is forced externally, the large amount of water in the three-dimensional network can effectively dissipate the external energy, which makes the degradable gel with compression resistance. The compressive strength of the degradable gel increases with the increase of temperature, and the stress corresponding to 50% compressive strain of the degradable gel prepared at 130 °C reached 0.25 MPa. During the molding process of the degradable gel, the increase of temperature can provide more energy for the rapid decomposition of the ammonium persulfate, which leads to the increase of crosslinking points and the increase of crosslinking density of the internal network, and thus improves the compressive performance of the degradable gel.

### 2.7. Plugging Performance and Core Damage Performance of Degradable Gel

The results of the plugging experiments and core damage experiments with degradable gel are shown in Table 2. From the experimental results, it can be seen that as the permeability of the sand-filled tube decreases from 2794.1 mD to 538.2 mD, the plugging pressure of the degradable gel gradually increases from 17.8 MPa to 25.2 MPa. The plugging pressure of the degradable gel in sand-filled tubes with different permeabilities is more than 15 MPa, indicating that the prepared degradable gel has good plugging performance. For the sand-filled tubes with different permeability, the core damage rate is below 5%, which is less damaging to the reservoir. Degradable gel can be degraded to a low viscosity liquid after the plugging is completed, substantially restoring the permeability of the porous medium to its initial value. This is due to the hydrolysis of the amide groups in the degradable gel structure at high temperatures, leading to gel degradation and minimal damage to the core. By blocking the formation pressure well and simultaneously protecting the reservoir, degradable gel serves a dual purpose.

### 2.8. Degradation Properties

#### 2.8.1. The Degraded Residual Liquid Viscosity

**Figure 7:**
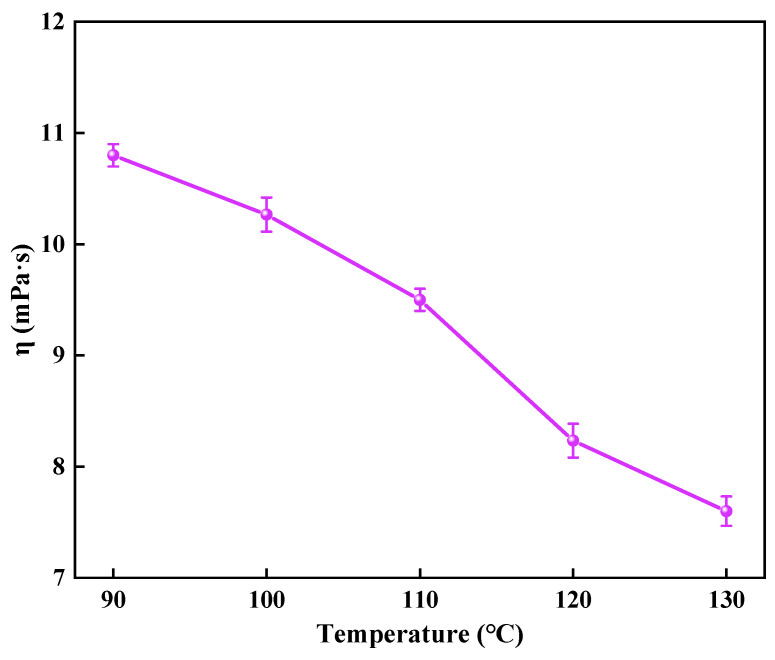
The residual viscosity of degradable gel after degradation.

The residual liquid after complete degradation of the gel is a Newtonian fluid. The viscosity of the residual liquid was further tested, and the results are shown in Figure 7. The degradable gel can automatically degrade to a low viscosity fluid after plugging, and the viscosity of the degraded residual liquid is less than 11 mPa·s, and the residual liquid can be discharged out of the wellbore with the formation fluid. As the temperature increases, the viscosity of the residual liquid is also reduced. The increase in temperature intensifies molecular thermal movement, increasing the likelihood of collision between water molecules and amide groups on the unstable crosslinker in the gel. This results in an accelerated hydrolysis rate of the amide groups, breakage of crosslinking points, destruction of structural stability, and the transformation of the degradable gel from a solid state to a low viscosity liquid. On the other hand, the quaternary ammonium cation on the γ-position carbon atom of the amide group has a certain promotion effect on the hydrolysis of the amide group. Due to the use of unstable crosslinkers, the gel can maintain its high strength for a period of time before gradually undergoing self-breaking. This transition results in the gel losing its high strength and transforming into a liquid with lower viscosity. This process helps to prevent further gel breaking, ultimately saving costs and simplifying the construction process.

#### 2.8.2. SEM Analysis

**Figure 8:**
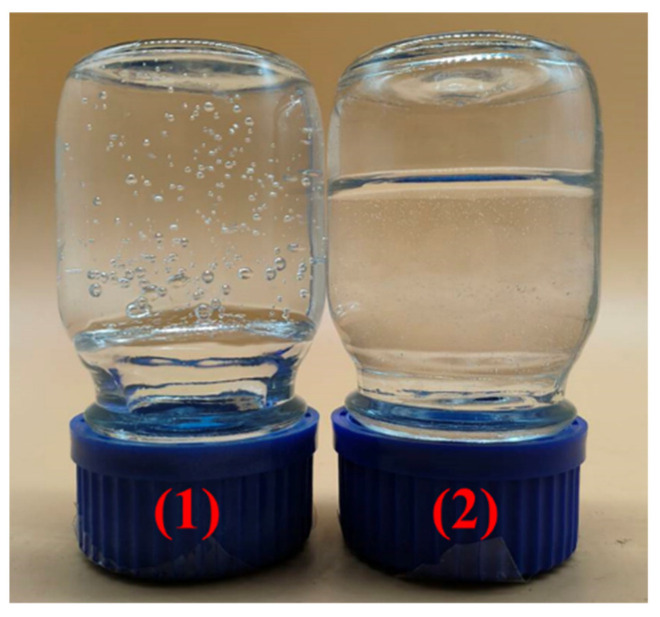
The picture of degradable gel: (1) after gelation, (2) after degradation.

**Figure 9:**
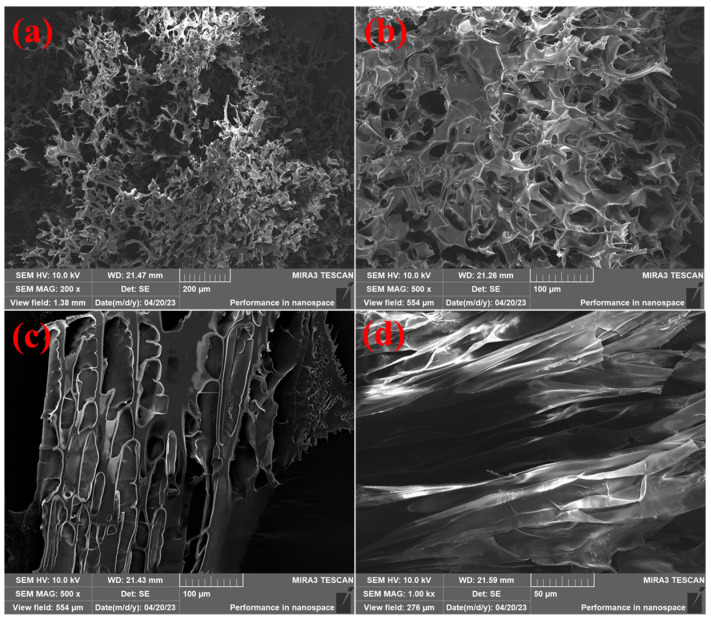
Micromorphology of degradable gel: (**a**,**b**) before degradation; (**c**,**d**) after degradation.

**Figure 10:**
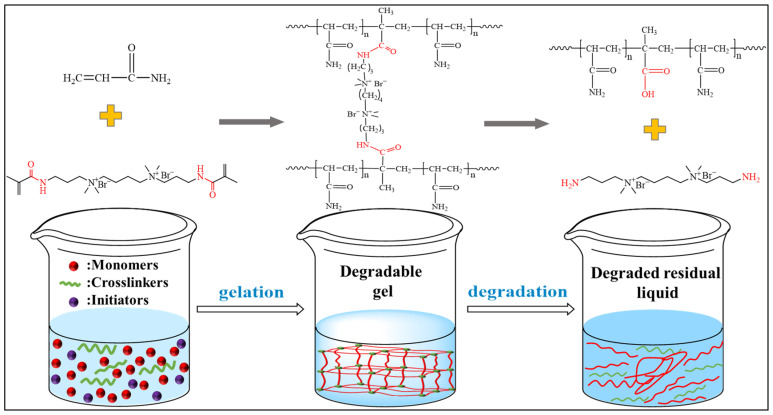
Schematic degradation mechanism of the degradable gel.

Figure 8 shows the physical picture of the degradable gel. The degradable gel loses its fluidity after gelation and turns into a transparent solid-like substance. When the reagent bottle was inverted, the surface of the gel did not deform, and its strength was evaluated at the grade of I. After complete degradation, the degradable gel changed from a solid-like substance to a liquid with a certain viscosity and good flowability. Scanning electron microscope (SEM) was used to observe the microscopic morphology of the samples before and after the degradation of the gel. The results are presented in Figure 9, where Figure 9a,b display the microscopic morphology of the degradable gel after gelation at different magnifications. The bulk of the degradable gel structure is composed of a large number of dense network structures, while the gaps between the network structures are small and the structure is relatively tight. Magnified to 500 times, there are also tiny filamentary crosslinking structures between the net structures, which crosslink each other between the layers. The pores of the degradable gel are originally filled with a large amount of water. This water sublimated out of the gel during the pretreatment step of freeze-drying, which led to the formation of the network structure. This structure is filled with a large amount of water, which gives it a strong water-holding capacity, good stability, exhibits high mechanical properties and flexibility, and can withstand formation pressure. Figure 9c,d show the microscopic morphology of the degradable gel after complete degradation at different magnifications. It is obvious that the degradable gel no longer possesses a dense structure after degradation has occurred. The structure collapses and transforms into a folded laminar structure, and the structure is hardly observed. Figure 10 illustrates the degradation mechanism of the degradable gel. The reason for this phenomenon is that the amide structure in the crosslinker is unstable, which can be hydrolyzed under the high temperature, leading to the breakage of the crosslinking point, and the reduction of the crosslinking density leads to the destruction of the structure, and the strength of the degradable gel decreases, and it gradually changes from a viscoelastic solid to a low-viscosity liquid.

In conclusion, the degradable gel synthesized in this study demonstrated favorable properties. Unlike conventional plugging gels, this gel can degrade autonomously following a period of pressurized operation, allowing it to be expelled back into the ground without the need for a gel-breaking agent, thereby minimizing harm to the formation. Furthermore, in contrast to degradable gels formulated by researchers utilizing crosslinkers with ester bonds, the gel developed in this study shows slower degradation at temperatures above 100 °C.

## 3. Conclusions

In this study, a degradable gel is prepared by using N, N′-butylbis[(3-acrylamidopropyl) dimethylammonium bromide] as crosslinker for temporary plugging in high-temperature reservoir. The higher the temperature, the shorter the degradation time for the gel. By varying the amount of crosslinker, the degradation time can be adjusted within the range of 90–130 °C. Increasing the concentration of crosslinker creates more crosslinked structures, improving the thermal stability of the degradable gel. The degradable gel demonstrates good viscoelasticity and compressive performance. In sand-filled tubes with permeabilities ranging from 538.2 mD to 2794.1 mD, the sealing pressures of degradable gel are all above 15 MPa, while the core damage rates are less than 5%. After the plugging operation, the amide groups on the crosslinker molecules are hydrolyzed, leading to the collapse of the three-dimensional network structure. Consequently, the degradable gel automatically degrades into a low-viscosity residual liquid, avoiding the need for subsequent gel breaking, reducing costs, and simplifying the construction process.

## 4. Materials and Methods

### 4.1. Materials

Acrylamide, dimethyl aminopropyl methacrylamide (DMAPAM), phenothiazine, and 1,4-dibromobutane were analytically pure and were purchased from Shanghai Macklin Biochemical Technology Co., Ltd., Shanghai, China. Ammonium persulfate, sodium bisulfite, ether and ethanol were also analytically pure and purchased from Sinopharm Chemical Reagent Co., Ltd., Shanghai, China. Deionized water was prepared by the laboratory.

### 4.2. Synthesis of the Crosslinker

**Figure 11:**
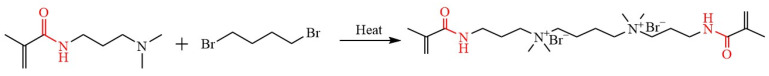
Equation for the high temperature unstable crosslinker.

Initially, 10.2 g of DMAPAM (0.06 mol) was dissolved in 10.2 g of ethanol. Subsequently, 4.32 g of 1,4-dibromobutane (0.02 mol) and 0.02 g of phenothiazine (0.01 mmol) were added to the solution. The reaction mixture was then stirred at 45 °C for a duration of 24 h. The product was precipitated with anhydrous ether and washed three times, and then dried in vacuum to obtain the high temperature unstable crosslinker. Figure 11 shows the reaction formula for the synthesis of unstable crosslinker.

### 4.3. Preparation of Degradable Gel

Firstly, a specific quantity of AM and the synthesized crosslinker were dissolved in deionized water, stirred thoroughly. Subsequently, a small portion of initiator was introduced, utilizing ammonium persulfate and sodium bisulfite as the redox initiation system. The mixture was continuously stirred before being injected into an ampoule. The sealed ampoule was then heated in a constant temperature oven, resulting in the formation of degradable gel after the solution had solidified completely. In order to study the effect of each preparation condition on the properties of the degradable gel, a four-factor, three-level orthogonal experiment was designed with monomer concentration, crosslinker concentration, initiator concentration, and temperature as the four factors, and the degradation time of the degradable gel as the response value. Combining the results of the literature review, the values of each factor were determined [14,22]. They cover the real field conditions. The orthogonal experiment design factors and levels are shown in Table 3.

### 4.4. Characterization and Performance Evaluation

#### 4.4.1. Characterization Tests

(1) Fourier Transform Infrared Spectroscopy (FTIR)

The Nicolet6700 fourier transform infrared spectrometer (Thermo Scientific, Waltham, MA, USA) was used to characterize the chemical structures of the crosslinker and degradable gel. The crosslinker and gel were freeze-dried and pulverized to obtain a white powder, and then the samples for testing were prepared by potassium bromide compression method. Test conditions: the scanning wave number range was 4000~500 cm^−1^ and the number of scans was 16 times.

(2) ^13^C NMR measurement

The ^13^C NMR spectrum of degradable gel was measured by BRUKER AVANCE NEO 400 WB NMR spectrometer (Bruker, Billerica, MA, USA). Solid-state NMR experimental parameters were set up: wide-cavity solid-state NMR spectrometer, 4 mm HXY triple resonance MAS probe, ^1^H and ^13^C resonance frequencies of 400.33 and 100.67 MHz, respectively. In the experiments, the RF field strength of the ^13^C channel ranged from 60 to 80 kHz, and that of the proton channel ranged from 45 to 90 kHz. The ^13^C NMR spectra were acquired using the cross-polarization (CP/MAS) method.

(3) Thermal Analysis

The degradable gel was cut into pieces and dried in an oven (50 °C) for 48 h. About 10 mg of the sample was weighed to test the thermal stability of the gel using a simultaneous thermal analyzer (Setaram, Lyon, France), which was heated from 30 °C to 600 °C in a nitrogen atmosphere at a rate of 10 °C/min.

(4) Scanning electron microscope

The degradable gel was freeze-dried and then fixed on the sample stage with conductive tape for gold spraying. Field emission scanning electron microscopy (TESCAN, Brno, Czech Republic) was used to observe the micro-morphology of the gel with an accelerating voltage of 10 kV.

#### 4.4.2. Performance Evaluation of Degradable Gel

(1) Measurement of gelation time and degradation time

The prepared degradable gel solution was injected into the ampoule at room temperature and the sealed ampoule was placed in an oven at 90–130 °C for complete reaction. The gelation time and degradation time were rapidly determined using the Sydansk gel code method [33]. In this study, the gelation time was defined as the time taken for the degradable gel to reach grade H (slightly deformable non-flowing gel), while the degradation time was defined as the time taken to degrade from grade H to grade C (flowing gel).

(2) Viscoelastic properties test

A HAAKE MARS 60 rheometer (Thermo Scientific, Karlsruhe, Germany) was utilized to analyze the viscoelastic characteristics of the degradable gel, specifically measuring the storage modulus G′ and the loss modulus G″. The oscillatory scanning test was carried out with a fixed angular frequency at 10 rad/s, a shear stress range of 0.1–1000 Pa, and a test temperature of 90 °C. To ensure reproducibility of the results, all tests included a pre-shear period before the official start of the test, followed by a sufficient rest period.

(3) Compressive strength test

The degradable gel reaction solution was loaded into a mold, and then prepared into a cylinder with a diameter of 25 mm and a height of 40 mm under the condition of 90–130 °C. The compression performance of the gel was tested by using WDW-100 electronic pressure tester (Bairoe, Shanghai, China) with a compression rate of 2 mm/min. When the degradable gel was compressed to the position of half of the original height, the size of the corresponding pressure value was recorded to obtain the compression stress-strain curve.

(4) Plugging performance test and core damage performance test

The plugging performance of the degradable gel was tested using a sand-filled tube displacement experiment. The initial permeability ($K_{1}$) of different sand-filled tubes was tested by injecting water with an advection pump at a rate of 0.5 mL/min. One pore volume (PV) of the degradable gel raw material liquid was injected into the sand-filled tubes. Then, the sand-filled tubes were removed, the inlet and outlet ends were sealed, and the tubes were placed in an oven at 90 °C for a reaction period of 2 h. Finally, the tubes were taken out of the oven, and the plugging performance was tested by injecting water into the tubes at a rate of 0.5 mL/min. During this process, pressure changes and liquid leakage at the outlet end of the tubes were continuously recorded. The maximum pressure before leakage was used as the plugging pressure of the degradable gel. After measuring the plugging pressure, the sand-filled tubes were again sealed at both ends and placed in a 90 °C oven. After one month, the permeability ($K_{2}$) of the sand-filled tubes were measured. The core damage rate P was calculated according to Equation (4).

(4) $$P=\left(\frac{K_{1}-K_{2}}{K_{1}}\right)\times 100\%$$

(5) Measurement of degraded residual liquid viscosity

The degradable gel was degraded at different temperatures (90–130 °C) and then the viscosity of the degraded residual liquid was tested. The viscosity of the liquid was assessed at various temperatures using the DV3TLVCP cone-plate viscometer (Brookfield, Middleboro, USA) with a CPA-41Z rotor, and the shear rate was varied from 1 s^−1^ to 100 s^−1^.

## Figures and Tables

**Table 1:** Orthogonal experiment results.

Number	Monomer Concentration (wt%)	Initiator Concentration (wt%)	Crosslinker Concentration (wt%)	Temperature (°C)	Degradation Time (d)
1	8	0.01	0.5	90	15
2	8	0.02	1	110	10
3	8	0.03	1.5	130	9
4	10	0.01	1.5	110	14
5	10	0.02	0.5	130	5
6	10	0.03	1	90	19
7	12	0.01	1	130	7
8	12	0.02	1.5	90	25
9	12	0.03	0.5	110	8
$k_{1}$	11.333	12.000	9.333	19.667	-
$k_{2}$	12.667	13.333	12.000	10.667	-
$k_{3}$	13.333	12.000	16.000	7.000	-
R	2.000	1.333	6.667	12.667	-

**Table 2:** Experimental results of plugging performance test and core damage test.

Sand-Filled Tube Number	Initial Permeability $K_{1}$ (mD)	Plugging Pressure (MPa)	Post-Degradation Permeability $K_{2}$ (mD)	Core Damage Rate P (%)
A	2794.1	17.8	2685.4	3.89
B	1559.2	20.1	1489.5	4.47
C	1185.6	22.7	1137.9	4.02
D	746.3	23.4	710.9	4.74
E	538.2	25.2	511.7	4.92

**Table 3:** Orthogonal experimental design table.

Factor	Level		
	1	2	3
Monomer concentration (wt%)	8	10	12
Crosslinker concentration (wt%)	0.5	1	1.5
Initiator concentration (wt%)	0.01	0.02	0.03
Temperature (°C)	90	110	130

## Data Availability

The data presented in this study are openly available in article.
